# Cell-free fetal DNA as a non-invasive method using pyrosequencing in detecting beta-globin gene mutation: A pilot study from area with limited facilities in Indonesia

**DOI:** 10.3389/fped.2022.902879

**Published:** 2022-08-04

**Authors:** Ani Melani Maskoen, Nurul Setia Rahayu, Bremmy Laksono, Azzania Fibriani, Willyanti Soewondo, Johanes C. Mose, Edhyana Sahiratmadja, Ramdan Panigoro

**Affiliations:** ^1^Department of Oral Biology, Faculty of Dentistry, Universitas Padjadjaran, Bandung, Indonesia; ^2^Department of Biomedical Sciences, Faculty of Medicine, Universitas Padjadjaran, Bandung, Indonesia; ^3^Laboratory of Molecular Genetics, Faculty of Medicine, Universitas Padjadjaran, Bandung, Indonesia; ^4^Department of Biology, School of Life Sciences and Technology, Institut Teknologi Bandung, Bandung, Indonesia; ^5^Department of Pediatric Dentistry, Faculty of Dentistry, Universitas Padjadjaran, Bandung, Indonesia; ^6^Department of Obstetrics and Gynecology, Faculty of Medicine, Universitas Padjadjaran, Bandung, Indonesia

**Keywords:** beta-thalassemia, prenatal screening, cell-free fetal DNA, pyrosequencing, non-invasive

## Abstract

**Background:**

Thalassemia is a monogenic, autosomal recessive, inherited disorder of the red blood cells caused by mutations or deletions in the globin gene. Approximately 6–10% of the Indonesian population carries the β-globin gene mutation; however, premarital screening is rarely conducted, and antenatal screening is optional. We explored the use of cell-free fetal DNA (cffDNA) as a potential non-invasive method of detecting the fetal β-globin gene mutation prenatally in pregnant women.

**Materials and methods:**

Pregnant mothers (*n* = 10), who were known carriers of thalassemia and who had a history of having borne a baby with thalassemia major, and their carrier husbands (*n* = 4) were recruited after providing consent. EDTA blood was drawn, and maternal DNA, including cffDNA, and paternal DNA were isolated. Maternal contamination tests were conducted using the variable number tandem repeat test for *ApoB* and *D1S80* loci. Allele quantification was performed by pyrosequencing. Known mutations from the bio-archived DNA of patients with thalassemia major (*n* = 16) were run alongside as a control.

**Results:**

In total, 7 out of 10 cffDNA successfully passed the maternal contamination test. The results of the allele quantification showed that six fetuses were predictive carriers of IVS1nt5 and one was predictive normal, in line with the allele quantification for the bio-archived DNA from patients with thalassemia major. The minimum threshold percentage for mutant A allele at cd26 was 32%, mutant T allele at IVS1nt1 was 23%, and mutant C allele at IVS1nt5 was 39%.

**Conclusion:**

Taking cffDNA from the mother’s blood proved useful as a non-invasive means of detecting the β-globin gene mutation using pyrosequencing allele quantification. This non-invasive method is of great interest for prenatal diagnosis in settings with limited facilities, as it minimizes the risk of abortion. Further study of other mutations of the β-globin gene is needed.

## Introduction

Thalassemia is a monogenic disorder of the red blood cells caused by a mutation or deletion in the globin genes, including α-, β-, γ-, or δ-globin; and β-thalassemia is the most prevalent type of thalassemia worldwide (1). Thalassemia, which is autosomal recessive, is inherited through Mendelian inheritance. There is a 1 in 4 chance that parents who are both carriers will have a child with β-thalassemia major (2). Although premarital screening for thalassemia is not performed in some countries, pregnant women can be screened for carrier status with a complete blood count during their first antenatal visit (3).

The World Health Organization has recommended prenatal diagnosis as a preventive action to control thalassemia in developing countries (4). Prenatal diagnosis is offered to at-risk couples to determine the status of the fetus, especially if the mother is a carrier or previously bore a child with thalassemia major. Chorionic villus sampling or amniocentesis is performed to obtain fetal material for molecular genetic analysis. In experienced hands, this invasive procedure should be accurate and safe; however, it is associated with a risk of spontaneous miscarriage (5). Therefore, it is thus necessary to develop safer methods of prenatal diagnosis for both the fetus and the mother.

A non-invasive method of prenatal diagnosis has been developed using cell-free fetal DNA (cffDNA) isolated from the simple peripheral blood plasma or the urine of pregnant women (6). cffDNA samples can be taken from 8 weeks of pregnancy. The concentration of cffDNA circulating in the mother’s plasma increases later during gestation. However, the small concentration of cffDNA is difficult to detect using conventional polymerase chain reaction (PCR)-RFLP or sequencing; thus, more highly sensitive detection techniques are required. Pyrosequencing is a real-time sequencing-by-synthesis method that enables rapid single nucleotide polymorphism analysis of short DNA sequences. The fact that this sensitive technique can quickly detect common β-thalassemia mutations (7) makes it of great interest in areas where facilities are limited.

Indonesia is located in the global thalassemia belt. It is estimated that approximately 6–10% of its more than 200 million inhabitants, or about 20 million people, are carriers of thalassemia (8). The western part of Indonesia has a spectrum of β-globin gene mutations, with the most common being IVS1nt5 (G > C), IVS1nt1 (G > T), and cd26 or HbE (G > A) (9). In the eastern part of the country, α-thalassemia predominates (10). However, no systematic nationwide carrier screening has yet been conducted. Our previous study, in a very limited area, showed that 6% of pregnant women with anemia had high HbA2, which indicates they were carriers of β-thalassemia (11). Therefore, in this study we explored the use of pyrosequencing with cffDNA to detect the common β-globin gene mutation in Bandung, western Indonesia, by allele quantification assay. Such a method might prove useful for non-invasive prenatal diagnosis in our under resourced area.

## Materials and methods

### Design of the study

This exploratory study involved pregnant women with a gestational age greater than 8 weeks. All women had previously borne a child with thalassemia major. After providing consent, these pregnant women and their husbands were asked to participate in the study. The study protocol was approved by the Ethical Committee of the Faculty of Medicine, Universitas Padjadjaran (no. 26/UN6.KEP/EC/2021). The study was conducted at the Molecular Genetics Laboratory of the Faculty of Medicine, Universitas Padjadjaran, in 2019 and 2020.

### Isolation of DNA parents and cell free fetus DNA

Venous blood samples were taken from pregnant women and their husbands, and collected in a 3 mL EDTA tube. The tube was centrifuged at 1,200 × *g* for 10 min at 4°C to separate the plasma from the red blood cells. The upper layer, which was the plasma, was transferred to a 1.5 mL tube and centrifuged further using a Norgren Plasma/Serum Cell-Free Circulating DNA Purification Mini Kit (Cat. No. 55100; Norgen Biotek, Canada) according to the manufacturer’s protocol. The plasma was centrifuged at 2,400 × *g* for 20 min at 4°C, as described previously (12), to isolate the cffDNA. Furthermore, the buffy coat layer was taken for isolation of maternal DNA using the homebrew method. The concentration of DNA was measured by spectrophotometer (Nanodrop 200; Thermo Scientific). As a positive control, we used archived DNA samples from patients with β-thalassemia major with known mutations (Sanger Sequencing), and this positive control was rerun using pyrosequencing to confirm the mutation.

### Variable number tandem repeat for maternal contamination tests

Maternal DNA as well as cffDNA were checked for VNTR by PCR to confirm whether the cffDNA obtained was from the fetus or otherwise contaminated with maternal DNA. PCR analysis was conducted with primers that amplified two gene loci: D1S80 and ApoB. The primers for D1S80 were 5′-GAA ACT GGC CTC CAA ACA CTG CCC GCC G-3′ (forward) and 5′-GTC TTG TTG GAG ATG CAC GTG CCC CTT GC-3′ (reverse), and the primers for ApoB were 5′-CCT TCT CAC TTG GCA AAT AC-3′ (forward) and 5′-ATG GAA ACG GAG AAA TTA TG-3′ (reverse), as published previously (13). The electrophoresis images showed the pattern of the DNA bands; cffDNA was not contaminated with maternal DNA when both DNA bands showed different patterns (figure not shown).

### Detection of β-globin mutation by pyrosequencing

**FIGURE 1:**
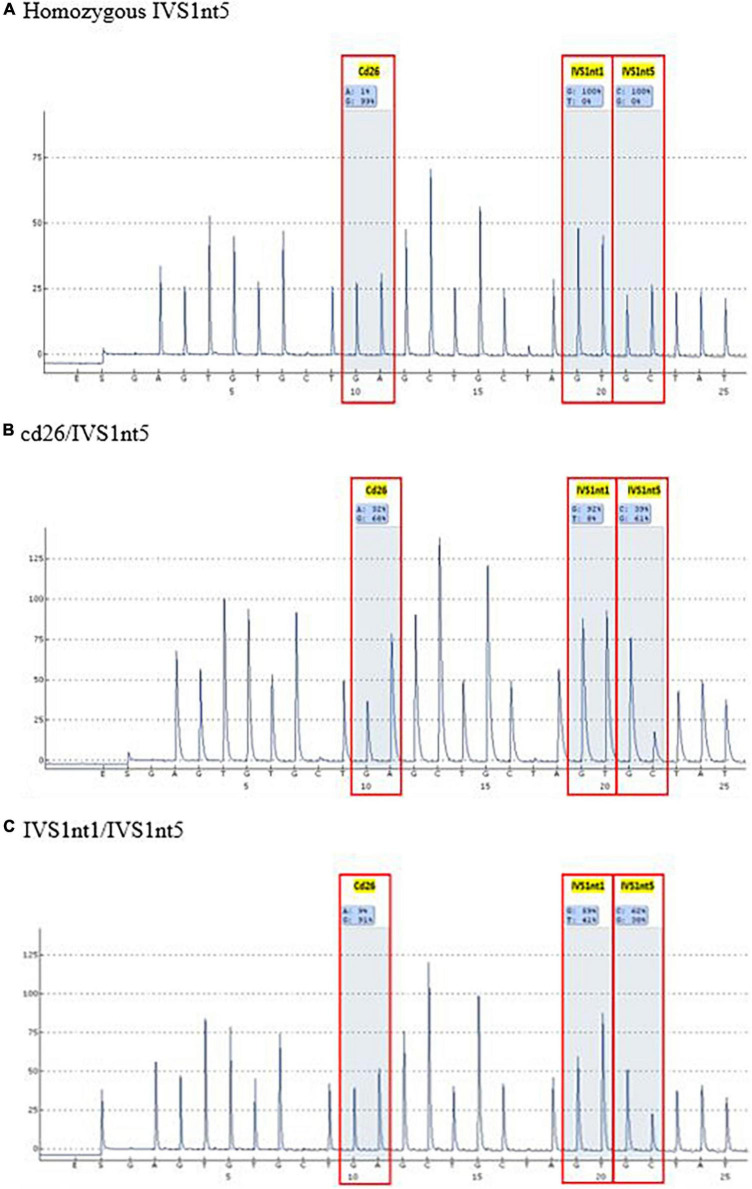
Pyrograms of controls positive for β-globin mutations for IVS1nt1, IVS1nt5, and cd26.

Before undergoing pyrosequencing, all samples were amplified with β-globin gene primers, resulting in a PCR product of 106 base pairs. Pyrosequencing was performed to target the HBB gene (hemoglobin subunit beta; GeneBank accession: NG_000007.3). This gene has three common mutations in Indonesia: IVS1nt5, IVS1nt1, and cd26. Pyrosequencing allele quantification was conducted using a primary sequences design (Pyrosequencing Assay Design Software) as follows: 5′-GGC AAG GTG AAC GTG GAT G-3′ (forward), 5′-biotin TGT CTC CAC ATG CCC AGT TT-3′ (reverse) and 5′-GGT GAA CGT GGA TGA-3′ (sequencing). The sequence to analyze for inclusion was AGT TGG TGG TRA GGC CCT GGG CAG KTT GST ATC AAG GTT ACA AGA CAG GTT TAA G (PyroMark Q96 v2.5.10). The results of the pyrosequencing allele quantification were shown in the form of pyrograms (PyroMark Q96 ID) in Figure 1, in which the target area appeared as a percentage of normal alleles or mutant alleles (Table 1). The samples presented in Table 1 were considered as positive controls. This samples were isolated DNA from buffy-coats and not from serum derived DNA samples, demonstrating the capacity of pyrosequencing to detect the mutation in patients or carriers. The type of mutation was first confirmed by the sequencing method, for example, a mutant A allele at cd26, or a mutant T allele at IVS1nt1, or a mutant C allele at IVS1nt5, then these samples were rerun using the pyrosequencing. The minimum percentage threshold in the pryrosequencing result was used to predict the normal or mutant alleles (14). The blood of baby at birth was further confirmed using pyrosequencing and a standard DNA examination by sequencing as previously described (15).

**TABLE 1:** The type of β-thalassemia mutations among thalassemia major patients as positive control using pyrosequencing.

Samples no.	Allele percentage (%)						Type of mutation*
	cd26 (G > A)		IVS1nt1 (G > T)		IVS1nt5 (G > C)		
	G	A	G	T	G	C	
Ctrl-1	56	44	100	–	30	70	cd26/IVS1nt5
Ctrl-2	99	1	63	37	38	62	IVS1nt1/IVS1nt5
Ctrl-3	97	3	77	23*^b^*	29	71	IVS1nt1/IVS1nt5
Ctrl-4	68	32*^a^*	92	8	61	39*^c^*	cd26/IVS1nt5
Ctrl-5	60	40	100	–	15	85	cd26/IVS1nt5
Ctrl-6	96	4	100	–	-	100	Homozygous IVS1nt5
Ctrl-7	99	1	100	–	-	100	Homozygous IVS1nt5
Ctrl-8	99	1	100	–	-	100	Homozygous IVS1nt5
Ctrl-9	100	–	100	–	-	100	Homozygous IVS1nt5
Ctrl-10	95	5	100	–	-	100	Homozygous IVS1nt5
Ctrl-11	61	39	100	–	46	54	cd26/IVS1nt5
Ctrl-12	60	40	100	–	38	62	cd26/IVS1nt5
Ctrl-13	67	33	96	4	50	50	cd26/IVS1nt5
Ctrl-14	100	–	91	9	–	100	Homozygous IVS1nt5
Ctrl-15	100	–	100	–	-	100	Homozygous IVS1nt5
Ctrl-16	97	3	100	–	-	100	Homozygous IVS1nt5

Ctrl, control: bio-archived DNA sample of thalassemia major patients as positive control. *Type of mutation as confirmed by sequencing method. The minimum threshold percentage for ^a^mutant A allele at cd26 was 32%; ^b^mutant T allele at IVS1nt1 was 23%, and ^c^mutant C allele at IVS1nt5 was 39%.

## Results

In total, 10 cffDNA were isolated (CFF-1 to CFF-10) along with 10 maternal DNA (SM-1 to SM-10) and four paternal DNA (SP-3, SP-7, SP-8, and SP-9). As a positive control, 16 bio-archived DNA of β-thalassemia patients with known mutations were used (Ctrl-1 to Ctrl-16). The highest concentration of DNA measured in maternal DNA was 130 ng/μL (SM-3 and SM-4), whereas, the lowest was 59 ng/μL (SM-2). As for cffDNA, the highest concentrations were 3 and 2.3 ng/μL for CFF-5 and CFF-7, respectively.

**FIGURE 2:**
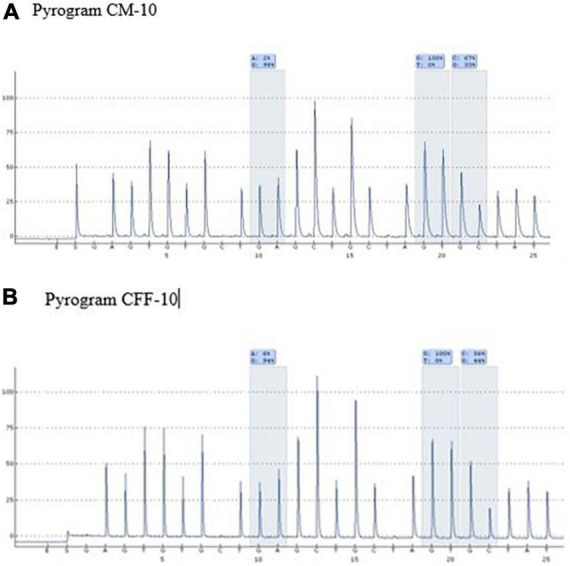
Pyrograms of maternal and fetal DNA (samples SM-10 and CFF-10, respectively) showing both as carriers of IVS1nt5.

A mutation at homozygous IVS1nt5 showed a 100% C allele or mutant allele, indicating a homozygous IVS1nt5 mutation. Of the cd26/IVS1nt5 mutations, 32–44% had an A allele at cd26 and 39–85% had a C allele at IVS1nt5. Of the IVS1nt1/IVS1nt5 mutations, 23–37% had a T mutant allele at IVS1nt1 and 62–71% had a C allele at IVS1nt5, all in concordance with the results of Sanger sequencing (Table 1). The minimum threshold percentage of each mutant allele in each phenotype was as follows: mutant A allele at cd26 = 32%, mutant T allele at IVS1nt1 = 23%, and mutant C allele at IVS1nt5 = 39%. For example, allele percentage in IVS1nt5 G > C for maternal (SM-10) and fetal DNA (CFF-10) were 67% and 56%, respectively (Figure 2), indicating that maternal dan fetal DNA were both carriers of IVS1nt5.

The cffDNA results showed that of the 10 cffDNA samples isolated, seven passed the maternal contamination test (Table 2) and were run together with the maternal and paternal DNA (Table 3), revealing six fetuses as predictive carriers of IVS1nt5 and one as a predictive normal fetus. The final outcome of the thalassemia status was further confirmed with DNA analysis after birth, resulting in a conformed predictive result in six babies as shown in Table 3. The babies were healthy grown up and had reached age of 2–3 years old. Interestingly, one case (CFF-4) was lost to follow up, with predictive allele percentage 44% for IVS1Nt5 carrier. This case had allele percentage 13% for IVS1nt1, leaving a question of a minimum threshold for mutant T allele at IVS1nt1 or otherwise compound heterozygote. However, in addition, two babies were re-examined using pyrosequencing at birth (BAB, baby at birth), showing a normal baby (BAB-5) and a carrier baby (BAB-7) for IVS1nt5 which were confirmed by DNA sequencing. Further study with more cases to confirm the minimum threshold was necessary.

**TABLE 2:** The contamination test result and the DNA pattern at locus *ApoB* and *DIS80* between cffDNA and maternal DNA.

cffDNA Sample no.	DNA pattern at locus		Conclusion
	*ApoB*	*D1S80*	
CFF-1	Same with mother	Same with mother	Contaminated
CFF-2	Same with mother	Same with mother	Contaminated
CFF-3	Different with mother	Same with mother	Pass
CFF-4	Different with mother	Same with mother	Pass
CFF-5	Different with mother	Same with mother	Pass
CFF-6	Different with mother	Same with mother	Pass
CFF-7	Different with mother	Different with mother	Pass
CFF-8	Same with mother	Same with mother	Contaminated
CFF-9	Different with mother	Same with mother	Pass
CFF-10	Different with mother	Same with mother	Pass

cff, cell free fetal; DNA, deoxyribose nucleic acid.

**TABLE 3:** Pyrosequencing results of parents and fetuses.

Samples	Allele percentage (%)						Predictive
	Cd26 (G > A)		IVS1nt1 (G > C)		IVS1nt5 (G > C)		Beta globin mutation
	G	A*^a^*	G	T*^b^*	G	C*^c^*	by pyrosequencing
SM-3	93	7	100	–	39	61	IVS1nt5 carrier
SP-3	97	3	100	–	40	60	IVS1nt5 carrier
CFF-3	98	2	100	–	38	62	IVS1nt5 carrier*^conf^*
SM-4	100	–	95	5	32	68	IVS1nt5 carrier
CFF-4	100	–	87	13	56	44	IVS1nt5 carrier*^n.d.^*
SM-5	100	–	100	–	22	78	IVS1nt5 carrier
CFF-5	96	4	100	–	79	21	Normal
BAB-5	87	13	100	–	84	16	Normal*^conf^*
SM-6	92	8	100	–	37	63	IVS1nt5 carrier
CFF-6	97	3	100	–	50	50	IVS1nt5 carrier*^conf^*
SM-7	100	–	100	–	15	85	IVS1nt5 carrier
SP-7	99	1	100	–	11	89	IVS1nt5 carrier
CFF-7	98	2	100	–	56	44	IVS1nt5 carrier
BAB-7	82	18	100	–	53	47	IVS1nt5 carrier*^conf^*
SM-9	94	6	100	–	37	63	IVS1nt5 carrier
SP-9	95	5	100	–	30	70	IVS1nt5 carrier
CFF-9	97	3	100	–	46	54	IVS1nt5 carrier*^conf^*
SM-10	98	2	100	–	33	67	IVS1nt5 carrier
CFF-10	94	6	100	–	44	56	IVS1nt5 carrier*^conf^*

SM, maternal DNA; SP, paternal DNA; CFF, fetal DNA; BAB, baby DNA at birth; conf., confirmed at birth by DNA examination; n.d., not determined. The minimum threshold percentage for ^a^mutant A allele at cd26 was 32%; ^b^mutant T allele at IVS1nt1 was 23%, and ^c^mutant C allele at IVS1nt5 was 39%.

## Discussion

According to the National Health Insurance in Indonesia, thalassemia major is listed as the fifth catastrophic disease (16), therefore, reducing the thalassemia major incidence with a movement toward zero thalassemia major is of great interest. However, the Health Law Article 75 allows abortion only for limited conditions and it is mandatory to have a counseling prior to and following the procedure (17). The abortion can only be performed in the first trimester of pregnancy, with consent of the woman and her husband (except rape victim). Moreover, the abortion procedure has to be conducted by health personnel who have expertise and authority, as well as being certified; and the health service facility needs to be appointed by the Ministry of Health. To prenatally diagnose fetus with thalassemia major, an invasive sampling has been employed. In some parts of Indonesia, where facilities for chorionic villus sampling are lacking, a very simple non-invasive method is ideal for prenatal diagnosis, especially for those at risk of having a baby with thalassemia major.

In this study, we developed a non-invasive method of using cffDNA to diagnose thalassemia prenatally, as chorionic villus sampling and amniocentesis are invasive and have a high risk of miscarriage. The wide-scale use of cffDNA to diagnose conditions prenatally has increased recently, as reviewed by Bianchi and Chiu (18). Although the concentration of cffDNA in the mother’s plasma is very small, our study shows that both maternal DNA and fetal DNA can be successfully isolated.

As expected, our study revealed a significantly higher concentration of maternal (SM) and paternal (SP) DNA (>50 ng/μL) extracted from blood compared to fetal DNA (<5 ng/μL). The low cffDNA concentration may be due to cracks in placental microparticles entering the mother’s blood during apoptosis (19). It is interesting that the concentration of cffDNA increases with gestational age (20). Hence, for the purpose of prenatal diagnosis, cffDNA needs to be taken mostly in the early trimester, or at about 8 weeks. The very low amount of cffDNA in the mother’s plasma is thus a critical point, and therefore a sensitive method of detection is needed (21). Techniques that are able to detect very low amounts of DNA include digital PCR and next-generation sequencing. However, these techniques are labor intensive and time consuming. The use of pyrosequencing allele quantification for non-invasive prenatal diagnosis might be a solution in areas where simple, quick, and inexpensive techniques are needed, especially in areas where thalassemia is prevalent (22).

The most common mutation among patients with thalassemia major in Indonesia is homozygous IVS1nt5 (9, 23). Our study detected the homozygous IVS1nt5 mutation in pyrograms with 100% allele C, which is a mutant allele, which indicates that the G allele had mutated completely into the C allele. Parents who carry thalassemia all have a low percentage of A or T mutant alleles compared to the threshold value for the thalassemia major control group; the percentage of the C mutant allele is > 39% (Table 3). Based on the low threshold value for the thalassemia major control group (Table 1), the parents are carriers of the IVS1nt5 mutation. Of seven cffDNA samples, six were predicted to be carriers of the IVS1nt5 mutation because they had a percentage of C alleles above a cutoff value of 39%. One cffDNA sample was predicted to be normal, with a percentage of mutant C alleles of 21% or below the threshold value.

Because of the high similarity between maternal and fetal sequences, detecting the fetal point mutation using cffDNA remains difficult. The cffDNA samples might have been contaminated with the presence of maternal DNA. Some techniques have been developed to determine maternal contamination, including those that use VNTR markers such as *ApoB* and *D1S80*. These two markers are widely used to diagnose various diseases, in forensics, and in paternity analysis (24, 25). However, the technique needs to be supported by the willingness of the fathers that need to be checked for their mutation. In this study, only four fathers were willingly tested and available out of 10 pregnancies. This might give a serious draw-back when checking the use of VNTR markers *ApoB* and *D1S80.* Furthermore, if pyrosequencing is an alternative for regular prenatal diagnosis, the higher chance of having contaminated samples (3 out of 10) need to be followed by a strategy. For example, it is preferable to perform a non-invasive pyrosequencing first and, if necessary, followed by the invasive chorionic villus sampling, since this invasive procedure harbors the risk of spontaneous abortion. Moreover, ethical concern may play an important issue in this prenatal diagnostic examination.

This study has several limitations, as it reports on the optimization stage of the development of a non-invasive sampling technique for detecting β-globin mutations. The threshold values of mutant alleles need to be determined, and more positive controls are needed. Furthermore, since the number of the sample size is small, more cases are needed. In addition, our limitation is that we do not confirm all positive results by pyrosequencing with standard DNA examination. Moreover, other new mutations, especially α-globin mutations, need to be curated in case of rare cases in an area.

## Conclusion

Cell-free fetal DNA by pyrosequencing serves as a non-invasive prenatal method and is of great use in areas with limited facilities, like Indonesia. The assay in this study has percentage limits of 32% for A allele, 23% for T allele, and 39% for C allele. However, further study is needed to confirm the results with more cases and to check after birth for confirmation.

## Data availability statement

The original contributions presented in this study are included in the article/supplementary material, further inquiries can be directed to the corresponding author.

## Ethics statement

The studies involving human participants were reviewed and approved by the Research Ethics Committee of Universitas Padjadjaran, Bandung, Indonesia (Ethical Clearance No. 26/UN6.KEP/EC/2021). The patients/participants provided their written informed consent to participate in this study.

## Author contributions

AM and ES designed the protocol and searched for the grant. NR had the master program Scholarship and helped working in the laboratory. AM, NR, and ES wrote the manuscript. All authors analysed the result, read and finalized the manuscript, and agreed to be accountable for the content of the work.

## Abbreviations

cffDNA: cell-free fetal DNA
Ctrl: bio-archived thalassemia major DNA samples as control group
EDTA: ethylenediaminetetraacetic acid
PCR: polymerase chain reaction
PCR-RFLP: polymerase chain reaction-restriction fragment length polymorphism
SM: maternal DNA samples
SP: paternal DNA samples
VNTR: variable number tandem repeat.
